# A long symmetric N⋯H⋯N hydrogen bond in bis­(4-amino­pyridinium)(1+) azide(1−): redetermination from the original data

**DOI:** 10.1107/S2056989017011537

**Published:** 2017-08-15

**Authors:** Jan Fábry

**Affiliations:** a Institute of Physics of the Czech Academy of Sciences, Na Slovance 2, 182 21 Praha 8, Czech Republic

**Keywords:** crystal structure, redetermination, hydrogen bonding, symmetric hydrogen bonds, refinement constraints, refinement restraints, Cambridge Structural Database

## Abstract

The structure of the title mol­ecular salt, C_10_H_13_N_4_^+^·N_3_^−^, has been redetermined from the data published by Qian & Huang [*Acta Cryst.* (2010), E**66**, o3086; refcode WACMIY (Groom *et al.*, 2016)]. The improvement of the present redetermination consists in a correction of the site-occupancy parameter of the bridging H atom between the pyridine rings, as well as of its position. The present study has shown that the bridging H atom (site symmetry 2) is involved in a symmetric N⋯H⋯N hydrogen bond, which is one of the longest ever observed [N⋯N = 2.678 (3) Å]. In addition, there are also present weaker N_am_—H⋯N_az_ hydrogen bonds (am = amine and az = azide) of moderate strength and π-electron pyridine⋯π-electron inter­actions in the structure. All the azide N atoms also lie on a twofold axis.

## Abstract 2

The redetermined structure of the title mol­ecular salt possesses one of the longest symmetric N⋯H⋯N hydrogen bonds known [N⋯N = 2.678 (3) Å].

## Chemical context   

**Figure 1:**
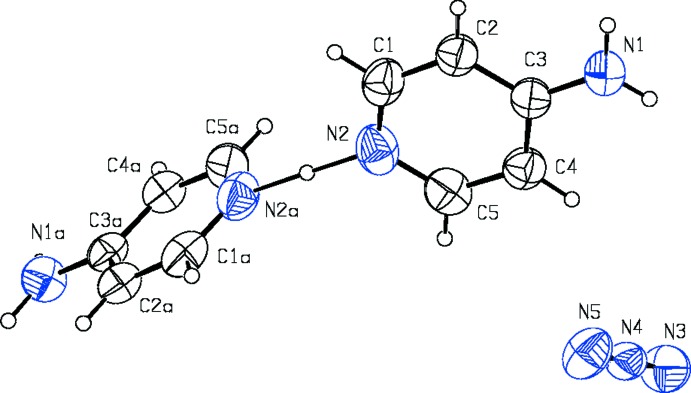
View of the constituent mol­ecules of the title structure after the improved refinement. The displacement ellipsoids are depicted at the 30% probability level (Spek, 2009 ▸).

Structures that contain hydroxyl and secondary and primary amine groups are sometimes determined incorrectly because of an assumed geometry of these groups from which the applied constraints or restraints were inferred. In such cases, the correct geometry is missed as it is not verified by inspection of the difference electron-density maps. Thus, a considerable number of structures could have been determined more accurately – *cf.* Figs. 1 ▸ and 2 ▸ in Fábry *et al.* (2014 ▸). The inclusion of such erroneous structures causes bias in crystallographic databases such as the Cambridge Structural Database (Groom *et al.*, 2016 ▸).
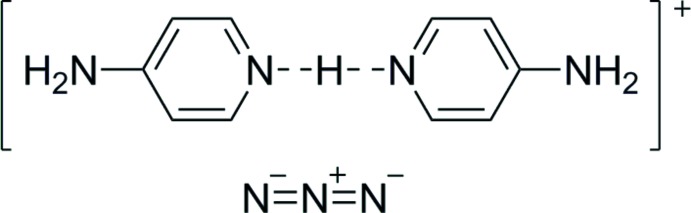


In the course of recalculation of suspect structures that were retrieved from the Cambridge Structural Database (Groom *et al.*, 2016 ▸), the structure determination of the title structure by Qian & Huang (2010 ▸) with the pertinent CSD refcode WACMIY became a candidate for a checking recalculation. The reason was that both the primary and secondary amine groups were constrained with distance constraints equal to 0.86 Å, with planar conformation and *U*_iso_(H) = 1.2*U*_eq_(N).

Inspection of the publication of the title structure by Qian & Huang (2010 ▸) has revealed that the bridging hydrogen atom H2*a*, lying between two symmetry-equivalent nitro­gen atoms related by a crystallographic twofold axis, was modelled by two (undisordered) H atoms both with occupational parameters equal to 1: such a structural motif is impossible. The present article describes the redetermination of bis­(4-amino­pyridinium)(1+) azide(1−), which was reported by Qian & Huang (2010 ▸).

## Structural commentary   

**Figure 2:**
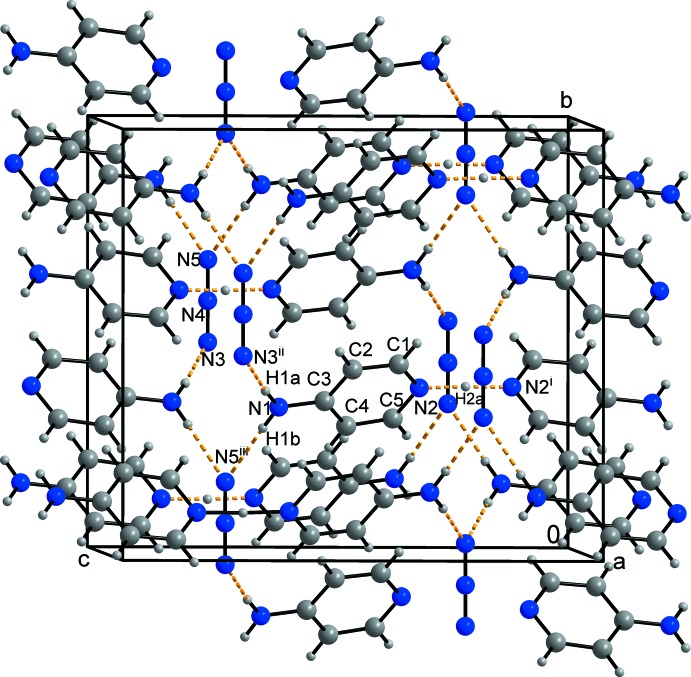
A view of the title structure along the unit-cell axis *a*. Symmetry codes: (i) −*x* + 1, *y*, −*z* + 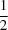; (ii) *x* + 1, *y*, *z*; (iii) *x* + 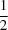, *y* − 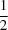, *z*. Applied colours for atoms: grey = C and H, blue = N; applied colours for bonds: black = covalent bonds, dashed orange = *H*⋯hydrogen bonds acceptor (Brandenburg & Putz, 2005 ▸).

The components of the title mol­ecular salt are shown in Fig. 1 ▸. It is seen that the bridging hydrogen atom (H2*a*) inter­connects symmmetry-related 4-amino­pyridine mol­ecules; the symmetry operation for atoms with the suffix ‘a’ is the same as symmetry code (i) in Table 1 ▸ and Fig. 2 ▸, *viz*. −*x* + 1, *y*, −*z* + 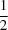. The inter­planar angle between the pyridine rings N2/C1–C5 and N2^i^/C1^i^–C5^i^ is 87.90 (7) °.

**Figure 3:**
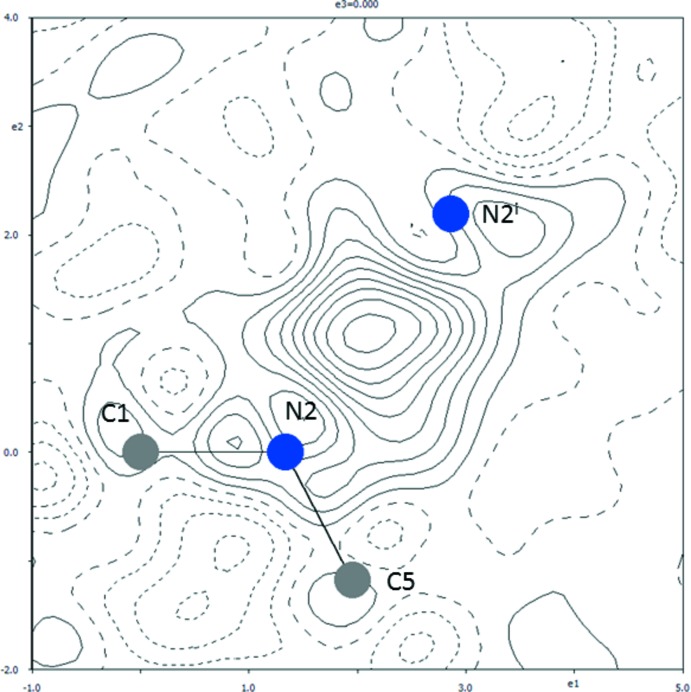
A section of the difference electron-density map for the present redetermined title structure, which shows the build up of the electron density between the atoms N and N^i^ [symmetry code: (i) −*x* + 1, *y*, −*z* + 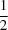]. Positive and negative electron densities are indicated by continuous and dashed lines, respectively. The increment of electron density between the neighbouring contours is 0.02 e Å^−3^ (Petříček *et al.*, 2014 ▸).

Table 1 ▸ lists the hydrogen bonds in the structure. The packing of the ions in the unit cell is shown in Fig. 2 ▸. Fig. 3 ▸ shows the difference electron-density map calculated without the bridging hydrogen atom H2*a* in the region N2⋯(H2*a*)⋯N2^i^. A well-defined, single peak in this map indicates that H2*a* is situated on a twofold axis, *i.e*. it is involved in a symmetric hydrogen bond while not being disordered. This hydrogen bond is the strongest hydrogen bond in the structure and is one of the family of long symmetric hydrogen bonds N⋯H⋯N as listed in Table 1 ▸. As Tables 1 ▸ and 2 ▸ show, the title structure contains the second longest known truly symmetric N⋯H⋯N hydrogen bond after CAFHAT01.

The remaining N—H_am_⋯N_az_ (am = primary amine, az = azide) hydrogen bonds are considerably weaker, though still of moderate strength (Gilli & Gilli, 2009 ▸). Atom H1*a* forms a link to the terminal azide nitro­gen atom N3 while H1*b* bonds to the other terminal azide atom N5. The graph-set motif is described in the *Supra­molecular features* section. In addition to the hydrogen-bonding inter­actions, there are also π-electron ring⋯π-electron pyridine inter­actions in the structure. The distance between the ring centroids N2/C1–C5 and N2^iv^/C1^iv^–C5^iv^ is 3.7145 (17) Å [symmetry code: (iv) −*x* + 1, −*y* + 1, −*z* + 1].

The primary amine group centered on N1 is almost planar [C3—N1—H1*a* = 120.0 (9), C3—N1—H1*b* = 119.1 (9), H1*a*—N1—H1*b* = 120.6 (13)°] despite the somewhat lengthened C3—N1 bond [1.345 (2) Å]. The reason may be found in the hydrogen bonds formed by the group with N—H⋯N bond angles being close to 180 °.

Once again, the present redetermination emphasizes the importance of careful examination of the difference electron-density maps during a structure determination.

## Supra­molecular features   

In addition to the above-mentioned symmetric hydrogen bond N2⋯H2*a*⋯N2^i^ [symmetry code: (i) −*x* + 1, *y*, −*z* + 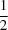] for which the graph-set motif notation is missing (the donors act simultaneously as acceptors in the title structure; Etter *et al.*, 1990 ▸) the principal graph-set motif in which the primary amine group as well the azide atoms are involved is 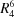
(20).

In a detail, the atoms involved in this graph-set motif are as follows (Fig. 2 ▸): N3^v^–H1*a* ^vi^–N1^vi^–H1*b* ^vi^–N5^ii^–N4^ii^–N3^ii^–H1*a*–N1–H1*b*–N5^iii^–H1*b* ^vii^–N1^vii^–H1*a* ^vii^–N3–N4–N5–H1*b* ^viii^–N1^viii^–H1*a* ^viii^ [symmetry codes: (ii) *x* + 1, *y*, *z*; (iii) *x* + 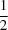, *y* − 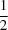, *z*; (v) *x* + 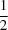, *y* + 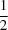, *z*; (vi) −*x* + 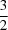, *y* + 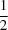, −*z* + 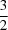; (vii) −*x* + 1, *y*, −*z* + 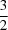; (viii) *x* − 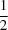, *y* + 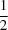, *z*].

The hydrogen bonds in this graph set motif are directed along the unit-cell parameter *b*.

## Synthesis and crystallization   

The preparation of the title compound was described by Qian & Huang *et al.* (2010 ▸) in the supporting information of their article.

## Database survey   

The structure determination by Qian & Huang (2010 ▸) has been included into the Cambridge Structural Database (Groom *et al.*, 2016 ▸) under the refcode WACMIY.

## Refinement   

Table 3 ▸ lists the details regarding the crystal data, data collection and the refinement. The starting structural model was taken from the determination by Qian & Huang (2010 ▸). All hydrogen atoms were discernible in the difference electron-density map. The aryl hydrogen atoms were constrained by C_ar­yl_—H_ar­yl_ = 0.93 Å and *U*_iso_(H_ar­yl_) = 1.2*U*_eq_(C_ar­yl_). The positional parameters of the primary amine hydrogen atoms were refined freely while their displacement parameters were constrained by *U*_iso_(H_N2_) = 1.2*U*_eq_(N2). The bridging hydrogen atom H2*a* involved in the symmetric hydrogen bond N2⋯H2*a*⋯N2^i^ was refined freely. Refinements using *JANA*2006 and *SHELXL* (Sheldrick, 2008 ▸) with the threshold for observed diffractions *I* = 2σ(*I*) led to the same result of the bridging hydrogen atom being located on the twofold axis.

## Supplementary Material

Crystal structure: contains datablock(s) global, I. DOI: 10.1107/S2056989017011537/hb7695sup1.cif

Structure factors: contains datablock(s) I. DOI: 10.1107/S2056989017011537/hb7695Isup2.hkl

Supporting information file. DOI: 10.1107/S2056989017011537/hb7695Isup3.smi

CCDC reference: 1566932

Additional supporting information:  crystallographic information; 3D view; checkCIF report

## Figures and Tables

**Table 1:** Hydrogen-bond geometry (Å, °)

*D*—H⋯*A*	*D*—H	H⋯*A*	*D*⋯*A*	*D*—H⋯*A*
N2—H2*a*⋯N2^i^	1.3391 (16)	1.3391 (16)	2.678 (3)	178 (2)
N1—H1*a*⋯N3^ii^	0.927 (14)	2.067 (14)	2.990 (2)	173.6 (13)
N1—H1*b*⋯N5^iii^	0.857 (16)	2.154 (16)	3.010 (2)	177.9 (14)

Symmetry codes: (i) 
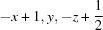
; (ii) 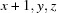; (iii) 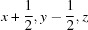.

**Table 2:** Structures with long N⋯H⋯N hydrogen bonds (Å, °) with a centred hydrogen For the search in the Cambridge Structural Database (Groom *et al.*, 2016 ▸), the *D*—H distance was set in the inter­val 1.30–1.45 Å and the non-bonding distance between the donor and acceptor nitro­gen atoms was set in the inter­val 2.6–3.0 Å.

Refcode	*D*—H	H⋯*A*	*D*⋯*A*	*D*—H⋯*A*
BOTXEO^*a*^	1.322 (3)	1.515 (3)	2.829 (4)	171.09 (16)
CAFHAT01^*b*^	1.34	1.37	2.7018	169.8
CAFHAT01^*b*^	1.35	1.35	2.7009	175.3
COFMUF10^*c*^	1.35 (10)	1.50 (10)	2.844 (7)	171 (11)
DAHGUO01^*d*^	1.33 (6)	1.38 (6)	2.690 (8)	168 (6)
EFAZOB^*e*^	1.32 (5)	1.38 (5)	2.692 (5)	176 (4)
EPIWU*X* ^*f*^	1.33 (3)	1.33 (2)	2.657 (9)	172 (8)
FISROP^*g*^	1.45 (4)	1.51 (4)	2.963 (3)	173 (2)
FOGKAP^*h*^	1.31 (4)	1.34 (4)	2.652 (5)	175 (4)
HUJNUW^*i*^	1.341 (15)	1.414 (16)	2.68 (2)	152.7 (8)
IYEVO*X* ^*j*^	1.33 (7)	1.37 (7)	2.691 (6)	174 (6)
MIJMUN^*k*^	1.27 (7)	1.56 (7)	2.812 (7)	165 (5)
MIJMUN^*k*^	1.34 (9)	1.52 (10)	2.808 (7)	159 (8)
OBUCOE^*l*^	1.33 (3)	1.43 (3)	2.736 (2)	165 (3)
QUHFEG^*m*^	1.39 (4)	1.40 (4)	2.792 (10)	176 (5)
SIZSUQ^*n*^	1.317 (14)	1.319 (14)	2.63 (2)	176.8 (9)
WOFGII^*o*^	1.33 (4)	1.39 (4)	2.706 (4)	167 (3)
XICRI*M* ^*p*^	1.31 (4)	1.52 (4)	2.826 (3)	164 (3)
ZEYLIA^*q*^	1.32 (4)	1.51 (4)	2.833 (4)	175 (3)

Notes: (*a*) 2-(1,3-Benzoxazol-2-yl)-1-phenyl­vinyl benzoate (Orozco *et al.*, 2009 ▸); (*b*) hydrogen bis­[bis­(2-{[(imidazol-4-yl)methyl­ene]amino}­eth­yl){2-[(imidazolato)methyl­ene]amino}­eth­yl)amine]­cobalt(III) triperchlorate hepta­hydrate (Marsh & Clemente, 2007 ▸); (*c*) 2,1,3-benzoselena­diazole 2,1,3-benzoselena­diazo­lium penta­iodide (Gieren *et al.*, 1985 ▸); (*d*) bis­{[1,4-diazo­niabi­cyclo­(2.2.2)octa­ne][1-aza-4-azoniabi­cyclo­(2.2.2)octa­ne]} tetra­kis­(tribromide) dibromide (Heravi *et al.*, 2005 ▸); (*e*) bis­[(3,5-di­methyl­pyrazole)(3,5-di­methyl­pyrazol­yl)]platinum(II) (Umakoshi *et al.*, 2008 ▸); (*f*) 4-{2-(pyridin-4-yl)­oxy]-1,2-bis­(2,3,5,6-tetra­fluoro-4-iodo­phen­yl)eth­oxy}pyridin-1-ium iodide bis­(nitro­benzene) (Martí-Rujas *et al.*, 2012 ▸); (*g*) 5,6:14,15-dibenzo-1,4-dioxa-8-azonia-12-aza­cyclo­penta­deca-5,14-diene 5,6:14,15-dibenzo-1,4-dioxa-8,12-di­aza­cyclo­penta­deca-5,14-diene per­chlorate (Tušek-Božić *et al.*, 2005 ▸); (*h*) dioxido­tetra­kis­(4-methyl­pyridine)­rhenium(V) 4-methyl­pyridinium 4-methyl­pyridine diodide (Krawczyk *et al.*, 2014 ▸); (*i*) 4-methyl­pyridinium *trans*-bis­(γ-picoline)tetra­kis­(thio­cyanato)­molybdenum 4-methyl­pyridine (Kitanovski *et al.*, 2009 ▸); (*j*) bis­(4,4′-bipyridinium) hexa­kis­(μ_2_-sulfido)­tetra­germaniumtetra­sulfide 4,4′-bi­pyridine hepta­hydrate (Wang *et al.*, 2003 ▸); (*k*) 4,4′-bipyridinium 4-(pyrid-4-yl)pyridinium 4,4′-bi­pyridine hexa­kis­(iso­thio­cyanato-*N*)-iron (Wei *et al.*, 2002 ▸); (*l*) tris­(2-benzimidazolylmeth­yl)ammonium 3,5-di­nitro­benzoate 3,5-di­nitro­benzoic acid clathrate (Ji *et al.*, 2004 ▸); (*m*) (2*R*,4*S*,5*R*)-9-(hy­droxy­imino)-6′-meth­oxy­cinchonan-1-ium (2*R*,4*S*,5*R*)-*N*-hy­droxy-6′-meth­oxy­cinchonan-9-imine chloride methanol solvate (Zohri *et al.*, 2015 ▸); (*n*) *catena*-[bis­(μ_2_-aqua)-(5-cyano-2*H*-1,2,3-triazole-4-carboxamide)(4-cyano-1,2,3-triazole-5-carboxamide)­sodium] (Al-Azmi *et al.*, 2007 ▸); (*o*) (1,1′-hydrogenbis{4-[1′-(4-pyrid­yl)ferrocen-1-yl]pyridine}) 4-[1′-(4-pyrid­yl)ferrocen-1-yl]pyridinium tris­(5-carb­oxy-2-thienyl­carboxyl­ate) bis­(thio­phene-2,5-di­carb­oxy­lic acid) (Braga *et al.*, 2008 ▸); (*p*) cytosinium 4-amino-2-hy­droxy­benzoate cytosine monohydrate (Cherukuvada *et al.*, 2013 ▸); (*q*) cytosinium acetyl­enedi­carboxyl­ate cytosine monohydrate (Perumalla *et al.*, 2013 ▸).

**Table 3:** Experimental details

Crystal data	
Chemical formula	C_10_H_13_N_4_^+^·N_3_^−^
*M*_r_	231.27
Crystal system, space group	Monoclinic, *C*2/*c*
Temperature (K)	291
*a*, *b*, *c* (Å)	7.507 (3), 12.247 (5), 13.634 (5)
β (°)	99.278 (5)
*V* (Å^3^)	1237.1 (8)
*Z*	4
Radiation type	Mo *K*α
μ (mm^−1^)	0.08
Crystal size (mm)	0.14 × 0.11 × 0.10

Data collection	
Diffractometer	Bruker SMART 1K CCD area-detector
Absorption correction	Multi-scan (*SADABS*; Bruker, 2000 ▸)
*T*_min_, *T*_max_	0.988, 0.992
No. of measured, independent and observed [*I* > 3σ(*I*)] reflections	3027, 1096, 787
*R*_int_	0.072
(sin θ/λ)_max_ (Å^−1^)	0.595

Refinement	
*R*[*F* > 3σ(*F*)], *wR*(*F*), *S*	0.034, 0.085, 1.48
No. of reflections	1096
No. of parameters	87
H-atom treatment	H atoms treated by a mixture of independent and constrained refinement
Δρ_max_, Δρ_min_ (e Å^−3^)	0.08, −0.07

Computer programs: *SMART* and *SAINT* (Bruker, 2000 ▸), *SHELXTL* (Sheldrick, 2008 ▸), *PLATON* (Spek, 2009 ▸), *DIAMOND* (Brandenburg & Putz, 2005 ▸) and *JANA2006* (Petříček *et al.*, 2014 ▸).

## supplementary crystallographic information

### Crystal data

**Table:**

C_10_H_13_N_4_^+^·N_3_^−^	*F*(000) = 488
*M**_r_* = 231.27	*D*_x_ = 1.242 Mg m^−^^3^
Monoclinic, *C*2/*c*	Mo *K*α radiation, λ = 0.71073 Å
Hall symbol: -C 2yc	Cell parameters from 1359 reflections
*a* = 7.507 (3) Å	θ = 3.0–25.4°
*b* = 12.247 (5) Å	µ = 0.08 mm^−^^1^
*c* = 13.634 (5) Å	*T* = 291 K
β = 99.278 (5)°	Block, colourless
*V* = 1237.1 (8) Å^3^	0.14 × 0.11 × 0.10 mm
*Z* = 4	

### Data collection

**Table:**

Bruker SMART 1K CCD area-detector diffractometer	1096 independent reflections
Radiation source: fine-focus sealed tube	787 reflections with *I* > 3σ(*I*)
Graphite monochromator	*R*_int_ = 0.072
φ and ω scans	θ_max_ = 25.0°, θ_min_ = 3.0°
Absorption correction: multi-scan (*SADABS*; Bruker, 2000)	*h* = −8→8
*T*_min_ = 0.988, *T*_max_ = 0.992	*k* = −12→14
3027 measured reflections	*l* = −16→15

### Refinement

**Table:**

Refinement on *F*^2^	18 constraints
*R*[*F* > 3σ(*F*)] = 0.034	H atoms treated by a mixture of independent and constrained refinement
*wR*(*F*) = 0.085	Weighting scheme based on measured s.u.'s *w* = 1/(σ^2^(*I*) + 0.0004*I*^2^)
*S* = 1.48	(Δ/σ)_max_ = 0.004
1096 reflections	Δρ_max_ = 0.08 e Å^−^^3^
87 parameters	Δρ_min_ = −0.07 e Å^−^^3^
0 restraints	

### Special details

**Table:**

Experimental. The structure was solved by direct methods (Bruker, 2000) and successive difference Fourier syntheses.

### Fractional atomic coordinates and isotropic or equivalent isotropic displacement parameters (Å^2^)

**Table:**

	*x*	*y*	*z*	*U*_iso_*/*U*_eq_	
C1	0.6719 (2)	0.44584 (12)	0.40174 (12)	0.0889 (6)	
H1	0.732036	0.496631	0.368331	0.1067*	
C2	0.72245 (17)	0.43524 (10)	0.50154 (11)	0.0778 (5)	
H2	0.814514	0.478469	0.535055	0.0934*	
C3	0.63529 (16)	0.35896 (9)	0.55351 (10)	0.0702 (5)	
C4	0.49858 (17)	0.29730 (11)	0.49826 (11)	0.0793 (5)	
H4	0.436698	0.245345	0.529429	0.0952*	
C5	0.4560 (2)	0.31347 (12)	0.39842 (12)	0.0936 (6)	
H5	0.364461	0.271568	0.362714	0.1123*	
N1	0.68183 (18)	0.34619 (10)	0.65226 (9)	0.0859 (5)	
H1a	0.776 (2)	0.3866 (12)	0.6868 (10)	0.1031*	
H1b	0.631 (2)	0.2960 (12)	0.6815 (11)	0.1031*	
N2	0.54018 (19)	0.38706 (11)	0.34930 (8)	0.0943 (5)	
N3	0	0.47492 (16)	0.75	0.1020 (8)	
N4	0	0.57130 (18)	0.75	0.0768 (6)	
N5	0	0.66663 (17)	0.75	0.1074 (8)	
H2a	0.5	0.389 (2)	0.25	0.160 (9)*	

### Atomic displacement parameters (Å^2^)

**Table:**

	*U*^11^	*U*^22^	*U*^33^	*U*^12^	*U*^13^	*U*^23^
C1	0.0946 (10)	0.0866 (10)	0.0930 (11)	0.0183 (8)	0.0376 (9)	0.0120 (8)
C2	0.0752 (8)	0.0760 (8)	0.0853 (10)	0.0109 (6)	0.0224 (7)	0.0054 (7)
C3	0.0685 (7)	0.0688 (7)	0.0762 (9)	0.0166 (6)	0.0205 (6)	0.0039 (6)
C4	0.0762 (8)	0.0795 (8)	0.0847 (10)	0.0065 (6)	0.0205 (7)	0.0007 (7)
C5	0.0948 (10)	0.0999 (10)	0.0854 (11)	0.0105 (8)	0.0125 (8)	−0.0094 (8)
N1	0.0909 (8)	0.0865 (8)	0.0804 (9)	−0.0012 (5)	0.0144 (6)	0.0093 (6)
N2	0.1093 (9)	0.1039 (9)	0.0725 (8)	0.0233 (7)	0.0229 (7)	0.0027 (7)
N3	0.0983 (12)	0.0857 (11)	0.1212 (15)	0	0.0154 (10)	0
N4	0.0625 (8)	0.1024 (13)	0.0667 (9)	0	0.0143 (6)	0
N5	0.1140 (14)	0.0918 (12)	0.1263 (15)	0	0.0495 (12)	0

### Geometric parameters (Å, º)

**Table:**

C1—H1	0.93	C5—H5	0.93
C1—C2	1.359 (2)	C5—N2	1.340 (2)
C1—N2	1.334 (2)	N1—H1a	0.927 (14)
C2—H2	0.93	N1—H1b	0.857 (16)
C2—C3	1.397 (2)	H1a—H1b	1.55 (2)
C3—C4	1.3935 (19)	N2—H2a	1.3391 (16)
C3—N1	1.345 (2)	N3—N4	1.180 (3)
C4—H4	0.93	N4—N5	1.168 (3)
C4—C5	1.362 (2)		
			
H1—C1—C2	118.36	C4—C5—H5	118.53
H1—C1—N2	118.36	C4—C5—N2	122.95 (13)
C2—C1—N2	123.27 (14)	H5—C5—N2	118.53
C1—C2—H2	120.21	C3—N1—H1a	120.0 (9)
C1—C2—C3	119.58 (12)	C3—N1—H1b	119.1 (9)
H2—C2—C3	120.21	H1a—N1—H1b	120.6 (13)
C2—C3—C4	116.91 (12)	C1—N2—C5	117.61 (13)
C2—C3—N1	121.21 (11)	C1—N2—H2a	123.9 (8)
C4—C3—N1	121.88 (12)	C5—N2—H2a	118.2 (9)
C3—C4—H4	120.16	N3—N4—N5	180.0 (5)
C3—C4—C5	119.68 (13)	N2—H2a—N2^i^	178 (2)
H4—C4—C5	120.16		

Symmetry code: (i) −*x*+1, *y*, −*z*+1/2.

### Hydrogen-bond geometry (Å, º)

**Table:**

*D*—H···*A*	*D*—H	H···*A*	*D*···*A*	*D*—H···*A*
N2—H2*a*···N2^i^	1.3391 (16)	1.3391 (16)	2.678 (3)	178 (2)
N1—H1*a*···N3^ii^	0.927 (14)	2.067 (14)	2.990 (2)	173.6 (13)
N1—H1*b*···N5^iii^	0.857 (16)	2.154 (16)	3.010 (2)	177.9 (14)

Symmetry codes: (i) −*x*+1, *y*, −*z*+1/2; (ii) *x*+1, *y*, *z*; (iii) *x*+1/2, *y*−1/2, *z*.
